# Retraction Note: A comprehensive explainable AI approach for enhancing transparency and interpretability in stroke prediction

**DOI:** 10.1038/s41598-026-47615-2

**Published:** 2026-04-07

**Authors:** Marwa El-Geneedy, Hossam El-Din Moustafa, Hatem Khater, Seham Abd-Elsamee, Samah A. Gamel

**Affiliations:** 1 Electronics and Communications Engineering Department, Faculty of Engineering, Mansoura University, Mansoura, 35516 Egypt; 2 Electrical Department, Faculty of Engineering, Horus University Egypt, New Damietta, 34518 Egypt; 3 Faculty of Engineering, Horus University Egypt, New Damietta, 34518 Egypt

Retraction of: *Scientific Reports* 10.1038/s41598-025-11263-9, published online 18 July 2025

The Editors have retracted this Article.

The Authors have used a publicly available dataset (The Stroke Prediction Dataset). Concerns have been raised regarding the provenance and validity of this dataset^1^. The Authors have been unable to provide any further information regarding the provenance or accuracy of the data included in this dataset. As this dataset was used for the training and validation of the proposed model in this Article, the Editors no longer have confidence in the reliability of the results and conclusions of this Article.

Hossam El-Din Moustafa and Samah A. Gamel agree with the retraction. Marwa El-Geneedy, Hatem Khater, and Seham Abd-Elsamee disagree with the retraction.
